# Ending student mistreatment: early successes and continuing challenges

**DOI:** 10.1080/10872981.2019.1690846

**Published:** 2019-11-30

**Authors:** Katherine T. Lind, Christina M. Osborne, Brittany Badesch, Alyssa Blood, Steven R. Lowenstein

**Affiliations:** a Department of Pediatrics, University of Colorado School of Medicine, Aurora, CO, USA; b Departments of Internal Medicine and Pediatrics, Johns Hopkins Hospital, Baltimore, MD, USA; c Department of Surgery, NY Presbyterian Hospital, Weill Cornell Medical Center, New York, USA; d Emergency Medicine and Medicine and Associate Dean for Faculty Affairs, University of Colorado School of Medicine, Aurora, CO, USA

**Keywords:** Medical education, medical student, mistreatment, learning environment, generational challenges, faculty accountability

## Abstract

**Problem:** Student mistreatment represents an ongoing challenge for US medical schools. Students experiencing mistreatment may become marginalized and cynical, and they have higher rates of burnout, depression and substance use disorders. Although numerous attempts to eliminate mistreatment have been proposed, best practices remain elusive. We formed a unique student-faculty collaboration (the Ending Mistreatment Task Force) that allowed all voices to be heard and enabled identification of five interventions to reduce mistreatment.

**Intervention:** The EMTF developed and implemented five key interventions: 1) a shared mistreatment definition; 2) measures to increase faculty accountability, including adding professionalism expectations to faculty members’ contracts and performance reviews; 3) a Professionalism Office to respond promptly to students’ reports of mistreatment and provide feedback to faculty; 4) tools to help teachers provide authentic learning environments for students, while addressing generational misunderstandings; and 5) student-produced videos, helping faculty understand the impact of mistreatment as seen through students’ eyes.

**Context:** These interventions occurred at one medical school where mistreatment reports were consistently above national averages.

**Impact:** Over 6 years, the interventions helped reduce the rate of student-reported mistreatment by 36% compared with a 4% decline across all US medical schools.

**Lessons:** The collaborations between students and faculty helped each party identify unexpected misunderstandings and challenges. We learned that students want hard questions, although faculty are often afraid to challenge students for fear of offending them or being reported. We clarified differences between mistreatment and sub-optimal learning environments and openly discussed the pervasive opinion that ‘some’ mistreatment is important for learning. We also identified ongoing challenges, including the need to solicit residents’ perspectives regarding mistreatment and develop proper responses to disrespectful comments directed at patients, family and colleagues. The collaboration reinforced students’ and faculty members’ shared commitment to upholding a respectful learning and clinical care environment and ending mistreatment.

Medical student mistreatment represents an ongoing challenge for medical schools across the USA. Mistreatment includes offensive, harsh, or insulting speech as well as threats of physical contact, inappropriate physical contact, sexual harassment, humiliation of learners, and discrimination [1–5]. Medical students who experience mistreatment report higher rates of burnout, depression, anxiety, and substance use disorders. [1,5–13] Demeaning, humiliating, and disrespectful comments directed at students also contribute to cynicism and an erosion of humanistic values [12–18]. Additionally, in clinical settings, disrespectful and dehumanizing comments interfere with communication and erode trust among health-care team members, which threatens the quality of care and patient safety. [1,6,10,19–21]

Today, medical student mistreatment is widely recognized, and mistreatment rates are measured annually on the Association of American Medical Colleges

(AAMC) Graduation Questionnaire (GQ) [1,11,22,23]. In 2019, across all US medical schools, 40.4% of the students reported experiencing mistreatment at least once during medical school, [24] and reducing the rate of student mistreatment has become a priority for medical schools and for the Liaison Committee on Medical Education [19,25].

A variety of interventions to end mistreatment have been implemented, including institutional ‘zero-tolerance’ policies, safe-reporting mechanisms, oversight committees, resilience programs, and professional training for faculty and residents [9,14,20,23,26–30]. Still, mistreatment remains an ongoing problem, and best practices to end mistreatment remain elusive.

Eradicating mistreatment in medical education has proved challenging for several reasons, including generational misunderstandings and a persistent belief among clinical teachers that harsh treatment of medical students is an essential or unavoidable part of medical education [2,28,31–34]. Teachers may be unaware that establishing a respectful learning environment promotes high quality and safe patient care [1,6,10,19–21]. Equally important, more than 75% of the students nationally decline to report mistreatment, largely because ‘the incident did not seem significant enough’ or because they feared retaliation. [24] Lack of reporting means that teachers who have engaged in mistreatment, often unintentionally, may not receive feedback, remediation, or other corrective actions.

For many years, students at the University of Colorado School of Medicine (CUSOM) have reported student mistreatment at higher rates than the national average. To address this challenge, a student-faculty committee was created in 2012. This group, the ‘Ending Mistreatment Task Force’ (EMTF), reviewed data from surveys and focus groups and determined that students differed significantly in their understanding of what constituted mistreatment, were unwilling to report mistreatment when it occurred, were confident that if they did report, ‘nothing would happen,’ and did not sense that student mistreatment was a high priority for the faculty or administration. The group then developed and implemented interventions at the student and faculty levels with the goal of eliminating the mistreatment of students at the CUSOM. In this paper, we describe five separate interventions aimed at ending mistreatment, highlighting the advantages that accrue from a strong collaboration among students, faculty and administrative leaders; we also outline the challenges that remain.

## Interventions: the ending mistreatment project

**Figure 1.**
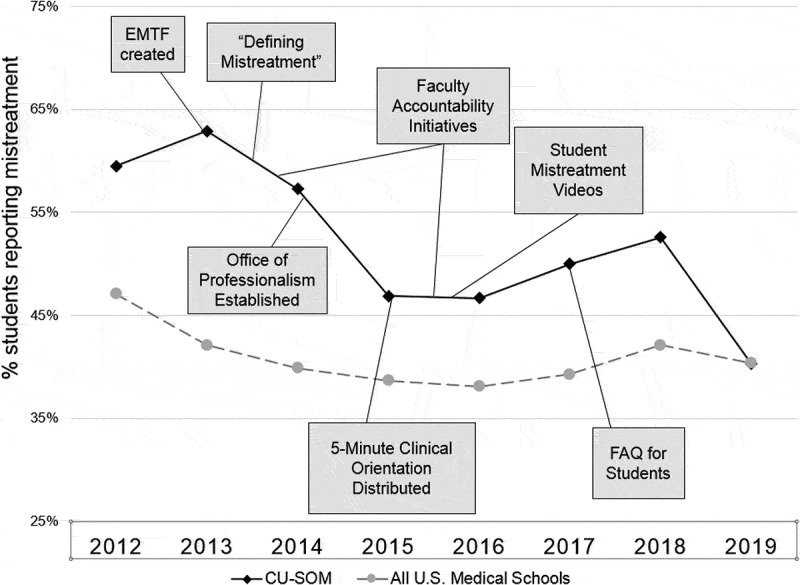
Timeline of interventions and reports of mistreatment from the AAMC Graduation Questionnaire. EMTF: Ending Mistreatment Task Force.Defining Mistreatment: A document providing definitions and examples to distinguish mistreatment from a sub-optimal learning environment.Faculty Accountability Initiatives: Included distribution of a new faculty Code of Professional Behavior, a revised Teacher-Learner Agreement, a Faculty Promise imbedded in annual faculty performance reviews and explicit description of professionalism obligations in letters of offer for new faculty.Office of Professionalism: A confidential resource for reporting mistreatment and a means to provide confidential, non-punitive feedback to teachers reported for mistreatment.The Office also conducted outreach activities in an effort to promote an institution-wide culture of professionalism and respect.*5-Minute Clinical Orientation*: A guide for clinical teachers that addressed myths about millennial learners, reinforced students' expectations for hard but respectful questions and recommended ‘best practices’ to help promote a respectful learning and clinical care environment.Student Mistreatment Videos: Student-produced videos illustrating examples of mistreatment, including student neglect, humiliating questioning, racial and gender bias toward students and dehumanizing remarks directed at patients or clinical care team members.FAQ for Students: A document produced by the Office of Professionalism and the EMTF, describing the procedures for reporting mistreatment and reinforcing the benefits and safety of reporting.

The Ending Mistreatment Task Force (EMTF) began as a collaborative effort between one student and one faculty member (CMO and SRL) but expanded to include student leaders from each medical school class and faculty and administrative representatives from the Offices of Student Life and Faculty Affairs, the CUSOM Academy of Medical Educators, the Senior Associate Dean for Education, the Assistant Dean for the Clinical Curriculum and the Office of Evaluation. Further, over a five-year period, the EMTF engaged with the student body, clinical clerkship directors, faculty governance leaders, hospital medical staff leaders and the CUSOM administration to develop and implement several interventions that sought to: define mistreatment; respond promptly and confidentially to students’ reports of mistreatment and other unprofessional behaviors; address generational misunderstandings; and empower stakeholders (including medical students, faculty leaders, clerkship directors, and administrators). An overriding goal of the EMTF was to reinforce teachers’ and learners’ shared commitment to a respectful learning environment and high-quality patient care. Here, we focus on five separate interventions deemed critical to ending mistreatment. These interventions were developed and refined after a comprehensive review of the literature as well as conversations and collaborations between members of the EMTF and the parties listed above. The interventions were implemented beginning in 2014 (Figure 1).

### Defining mistreatment

From the outset, it was apparent that medical students did not all define mistreatment in the same way. Harsh questions that were intimidating, humiliating, or marginalizing to one student were welcomed by another. Students who felt mistreated often were told by their peers that they are overly sensitive. Not surprisingly, students and faculty members also often defined mistreatment differently. In fact, faculty members often disengaged from discussions of mistreatment definitions, retreating to generic comments about soft and entitled ‘millennials.’

In the spring of 2013, the EMTF launched the ‘Defining Mistreatment’ project in order to promote a shared understanding of mistreatment among students, residents, and faculty members. The EMTF also sought to distinguish between mistreatment and the elements of a sub-optimal learning environment, including excessive shadowing, poor supervision, lack of authentic roles during clerkships, and ‘passive neglect’ by attending faculty members [3]. Although these behaviors may not constitute direct mistreatment, they can interfere with learning, compromise patient care, and marginalize students [5]. As one new fourth-year student told us, ‘Although I didn’t feel “mistreated” very frequently, there were [many] times that my learning environment was just mediocre … Not feeling like an important part of the clinical team was one of the most damaging experiences in education during my third year.’ These definitions were established after a thorough review of the existing literature, including the AAMC Graduation Questionnaire. [24] The examples of mistreatment and a sub-optimal learning environment, summarized in Tables 1 and 2, were reviewed by Medical Student Council and Faculty Senate leaders, leaders of the Office of Student Life, and clerkship directors prior to broad dissemination in the spring of 2014 to all students, residents, and faculty members.

**Table 1.** Examples of mistreatment defined by the ending mistreatment task force. ^a.^

Public belittlement or humiliation; Physical harm or the threat of physical harm; Requests to perform personal services; Being subjected to offensive, sexist remarks, or being subjected to unwanted sexual advances (physical or verbal); Being denied opportunities for training or rewards, or receiving lower evaluations or grades, based solely on gender; Being subjected to racially or ethnically offensive remarks; Being denied opportunities for training or rewards, or receiving lower evaluations or grades, based solely on race or ethnicity; Being subjected to offensive remarks about one’s sexual orientation; Being denied opportunities for training or rewards, or receiving lower evaluations or grades, solely because of sexual orientation; Verbal or emotional harassment through neglect or creating a hostile environment; Inappropriate comments about a student’s appearance; Use of foul language; Retaliation or threats of retaliation against any student who, in good faith, reports mistreatment or unprofessional behavior.

^a^ Adapted from: Dickstein L SM, Culbert A, Dobbins D, Hall F. *Appropriate Treatment in Medicine (ATM): A Compendium on Medical Student Mistreatment*. Washington, DC: Association of American Medical Colleges; 2000; and *Medical Student Graduate Questionnaire: 2017 All Schools Summary Report* Washington, DC: Association of American Medical Colleges; July 2017.

**Table 2.** Examples of a suboptimal learning environment ^a.^

Repeated lack of courtesy toward students, which may leave students feeling ignored or disenfranchised; Lack of courtesy or respect for patients or patient care team members^b^; Lack of clear learning objectives Excessive, repetitive “work without education” (the expectation that students perform an excessive number of tasks that are not related to teaching, learning or high-quality patient care or that are not appropriately supervised); Lack of teachers (faculty or residents) or insufficient time to ensure that education takes place during rounds or clinical encounters and that students receive constructive, performance-based feedback. Poor communication between teacher and student; Significant lack of attention paid to teaching and the role of learners, such that students are only allowed to “shadow,” rather than actively participate in patient care activities.

^a^ Adapted from: Gan R *et al*. When the learning environment is sub-optimal: exploring medical students perceptions of ‘mistreatment.’ Acad Med. 2014: 89:608–617.

^b^ May also properly be considered as an example of student mistreatment.

### Faculty engagement and accountability

The EMTF worked closely with CUSOM leaders, including the CUSOM Dean, the Associate Dean for Student Life, the Senior Associate Dean for Education, the Senior Associate Dean for Clinical Affairs, the Assistant Dean for the clinical curriculum, clinical clerkship directors, and the leaders of the Faculty Senate to identify steps to promote faculty ownership and accountability for ending mistreatment. First, it was necessary to develop a shared understanding of the causes and consequences of student mistreatment. After a comprehensive literature review, which was reinforced by open conversations between students and faculty members, the EMTF and Faculty Senate identified the following key contributors to student mistreatment:

- Entrenched hierarchies and power differentials that promote authoritarian teaching and clinical practice.
- Lack of continuity in clinical teams, because of short and ever-changing rotations.
- Clinical productivity pressures and high-stress clinical situations.
- Generational barriers (especially myths about millennials who ‘focus on work-life balance,’ ‘are afraid of tough questioning,’ or ‘do not appreciate the demands of clinical care’).
- A lack of confidential and non-punitive feedback to faculty members after minor and first-time professionalism lapses.
- The misguided belief that belittling comments on rounds reinforce learning.
- A lack of wellness and mental health support for faculty, residents, and others.

It became clear that many faculty members were also unaware of the consequences of student mistreatment, including public humiliation. Faculty members were afforded the opportunity to hear directly from students (both in-person and through the student-created videos described below) and to listen to presentations at Senate meetings and departmental grand rounds. After these experiences, faculty members expressed a deeper understanding that these behaviors not only interfere with learning, cause burnout, and potentiate mental health problems among medical students, but also undermine teamwork and threaten healthcare quality and patient safety [1,5–9,12].

Importantly, Faculty Senate leaders also acknowledged that, for more than 30 years, the faculty had not been sufficiently accountable for violations of the school’s professionalism standards. For example, professionalism standards and respect for learners, colleagues, and patients were not included in faculty contracts, annual performance reviews, nor at the time of review for promotion or tenure. As Brainard and Brislen (two medical students) posited, ‘the chief barrier to medical professionalism education [may be that] unprofessional conduct by medical educators is protected by an established hierarchy of academic authority’ [28]. Equally important, the CUSOM did not explicitly celebrate faculty members who demonstrated exemplary professionalism in their work.

Armed with new knowledge regarding the causes and consequences of medical student mistreatment, and working in collaboration with the deans of education, student life, clinical affairs and faculty affairs, the leaders of the faculty took ownership of their role in the problem:

- A new ‘Code of Professional Behavior’ was distributed, outlining critical faculty responsibilities and obligations.
- A ‘Faculty Promise’ was created and imbedded in faculty members’ annual performance reviews; by signing the Promise each year, faculty members recommit to upholding a positive learning environment and promoting respect and dignity for students, residents, colleagues, and patients, with no tolerance for mistreatment or retaliation against learners.
- The CUSOM ‘Teacher-Learner Agreement’ (TLA) was revised. The TLA summarizes students’ and teachers’ shared obligations to uphold a respectful learning environment. Importantly, the TLA states that teachers may ask challenging questions aimed at stimulating learning and self-discovery and identifying students’ knowledge gaps, but that they must avoid overly aggressive questioning that may be perceived as humiliating, degrading, or punitive.
- Explicit language regarding these professionalism obligations, including respect for learners, and links to the above-mentioned documents, were added to all employment contracts (letters-of-offer) for new faculty hires and appointment renewals.

### The 5-minute clinical orientation

Surveys performed as part of our student-faculty collaboration revealed several unexpected misunderstandings. First, students reported repeatedly that the faculty had become ‘hyper-aware’ of mistreatment. As one student observed, ‘our teachers seem so afraid of being disciplined for pimping us that they often choose to ignore us instead.’ Another student wrote, ‘I had several attendings joke, “Am I allowed to ask you questions? After faculty development, I don’t want to abuse you.”’ Secondly, as faculty members learned more about programs to promote student wellness and resiliency, some commented that this reflects students’ ‘innate weakness and mental fragility.’ One student reported, ‘Even attending physicians who care about us seem certain that we will break down in stressful clinical situations.’

In meeting with faculty teachers, students were given the voice to confront these cross-generational misunderstandings. Students emphasized that they are prepared for the stresses of the clinical environment and, while they do not wish to be mistreated, they are not ‘soft.’ The students reiterated that they desperately need hard questions that challenge them, teach them and help them grow [35,36]. One student reminded the task force of the saying, ‘strong sailors are not made on calm seas.’ The EMTF sought to reinforce the importance of maintaining a positive team environment that provides students a sense of meaning in their work while providing learning challenges [34]. Students also acknowledged that, while reporting a ‘sub-optimal learning environment’ is important, they are also prepared to accept responsibility for being sub-optimal learners. As one student explained, ‘When we are unprepared for rounds, we expect to be embarrassed.’ The EMTF then delivered the following message to CUSOM faculty members: *Students are prepared to work hard, stay late and make personal sacrifices in order to have a significant role in patient care*. This was done through meetings of the Faculty Senate, the clerkship directors, departmental Grand Rounds, the annual new faculty orientation program and the *5-Minute Clinical Orientation* document described below.

EMTF discussions highlighted that what is often missing in addressing mistreatment is the opportunity for faculty members and students to have timely conversations about their shared goals and expectations. To address this, we created the *5-Minute Clinical Orientation: Working with Your Students to Avoid Miscommunication and Mistreatment*, a brief teaching guide to encourage and enable faculty members to establish a culture of respect for learners, patients and colleagues. In this brief document, teachers are reminded to: set clear expectations for students at the start of each rotation; provide authentic patient care roles for students; ask challenging, patient care-related questions in a respectful way; refrain from making disrespectful comments directed at patients, other health-care professionals or team members; observe students in action and provide constructive feedback; acknowledge the gaps in knowledge that confront us all; and establish a supportive environment that encourages students to ‘speak up’ about ethical, professionalism, and patient care concerns. [10,29,37,38]

The 5-Minute Clinical Orientation guide focuses on positive steps that busy faculty members and residents can take to enhance communication, strengthen the learning environment, and avoid mistreatment. Further, the document directly addresses the generational divides and myths about younger generations of learners. A link to the guide is distributed annually to all clinical clerkship directors and teaching faculty, including new hires.

### Student-created mistreatment videos

The EMTF student leaders also prepared four videos, illustrating student neglect, humiliating questioning (‘malignant pimping’), racial and gender bias toward students, and disparaging or dehumanizing remarks about patients or clinical care team members. These videos were presented during departmental grand rounds and during the new faculty orientation and were posted online. The videos helped explain mistreatment and its impact through students’ eyes.

### Office of professionalism

As part of a coordinated effort to address learner mistreatment, the CUSOM established the Office of Professionalism (OOP) in 2014 in order to provide a safe and confidential resource for reporting episodes of mistreatment and other unprofessional behavior*s*. From its inception, the OOP was led by a senior academic physician with experience as a mediator and ombudsman and an academic clinical psychologist experienced in remediation and counseling.

The OOP was designed to respond professionally, confidentially, compassionately and quickly (within 24 hours) to reports of mistreatment or other unprofessional behaviors. Potential reporters include medical or graduate students, residents, clinical or post-graduate fellows, faculty members, administrators, and staff. The OOP was designed to respond to each investigation in a careful and confidential manner in an effort to verify reports, ascertain context, ensure the confidentiality and safety of reporters, prevent retaliation or the fear of retaliation, and ensure confidential, fair, and consistent treatment of faculty members and residents who have been reported.

After a first-time minor offense, the OOP may provide confidential and non-punitive feedback to help an offender gain insight and self-correct, thus reducing the likelihood of recidivism. A non-punitive feedback session also provides an opportunity for the offender to address conditions like stress, frustration, or burnout that may have contributed to the event.

When more serious or repeated offenses occur, the OOP team may recommend more directed interventions, such as coaching, training, remediation, diagnostic evaluation, or behavioral or substance use counseling. After serious transgressions, the OOP may recommend disciplinary actions to a faculty member’s department chair or another supervisor.

Primary prevention is also a critical component of the OOP’s mission, and OOP leaders seek to promote an institution-wide culture of compassion and respect through presentations at departmental grand rounds, participation at the annual orientation for new faculty, sharing of de-identified data, and other outreach activities. The OOP also works closely with clinical service and medical staff leaders to address workplace stresses and environmental factors that contribute to burnout, professionalism lapses, disengagement, and learner mistreatment [8,39]. Finally, the OOP collaborates with the CUSOM Resiliency Council and peer-to-peer coaching program, which provides support to faculty members who may have acted unprofessionally as a result of stress, burnout or adverse patient care outcomes.

To encourage students to report mistreatment, the OOP and EMTF developed various outreach tools, including a ‘Frequently Asked Questions’ document that reinforced for students the benefits and safety of reporting mistreatment.

## Methods

To assesss the impact of the five interventions, the EMTF tracked the rates of medical student mistreatment over a six-year period. Information regarding student-reported mistreatment was gathered from the annual AAMC Medical School Graduation Questionnaire (GQ), a national survey distributed to graduates of all US medical schools accredited by the Liaison Committee on Medical Education (LCME). [24] This national benchmarking survey has been conducted since 1978, in an effort to ‘identify and address issues critical to the future of medical education and the well-being of medical students … [including] students’ experiences of mistreatment in the learning environment.’[24] In 2019, 19,933 graduating medical students from 142 U. S medical schools participated in the GQ. During the six-year study period, the GQ participation rate among all graduating medical students in the US ranged from 80% to 84%. At the CUSOM the participation rate ranged from 78% to 87%.

In this study, the principal outcome measure (‘student-reported mistreatment’) was based on a single GQ survey item which sums participants’ responses to 16 questions regarding mistreatment experiences. These mistreatment experiences include public humiliation, threats or actual physical harm, disrespectful remarks, and discrimination based on gender, sexual orientation, race or other personal characteristics. For the 16 mistreatment behaviors, students were asked to include behaviors by faculty, nurses, interns or residents, staff or others. The GQ also provided information about the following related topics: Whether students ‘know the procedures … for reporting the mistreatment of medical students;’ the sources of mistreatment (faculty members, interns or residents, nurses or other staff); whether students reported the mistreatment incidents they experienced; and their reasons for not reporting.

Data specific to CUSOM medical students were collected from the annual GQ ‘Individual School Report,’[24] which also included national benchmarking data from ‘all schools.’ We tabulated the University of Colorado results over the six-year study period as a measure of the impact of the interventions we implemented. The national GQ data served as a concurrent comparison group and helped to control for temporal trends and other threats to the internal validity of the observed results. This study was determined to be exempt from review by the Colorado Multiple Institutional Review Board.

## Results

**Figure 2.**
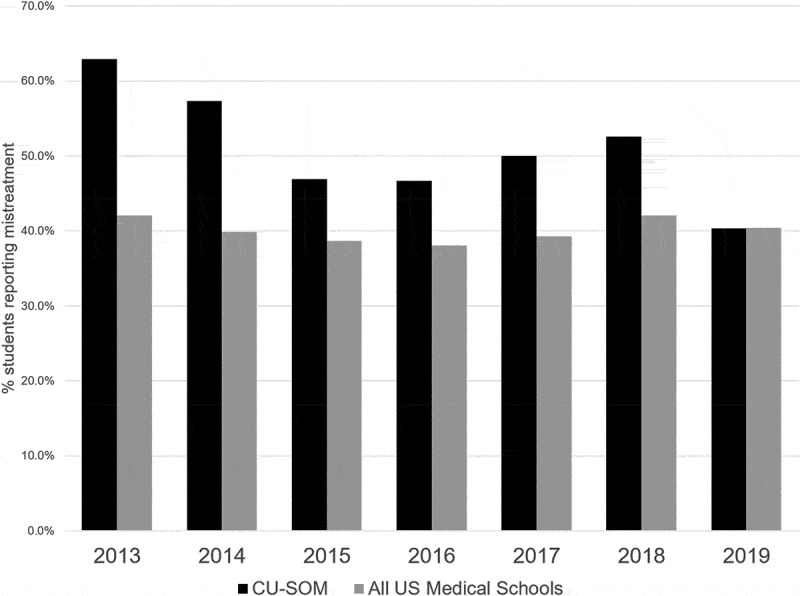
Rates of mistreatment – CU School of Medicine vs all US allopathic medical schools. Source: AAMC Graduation Questionnaire

**Figure 3.**
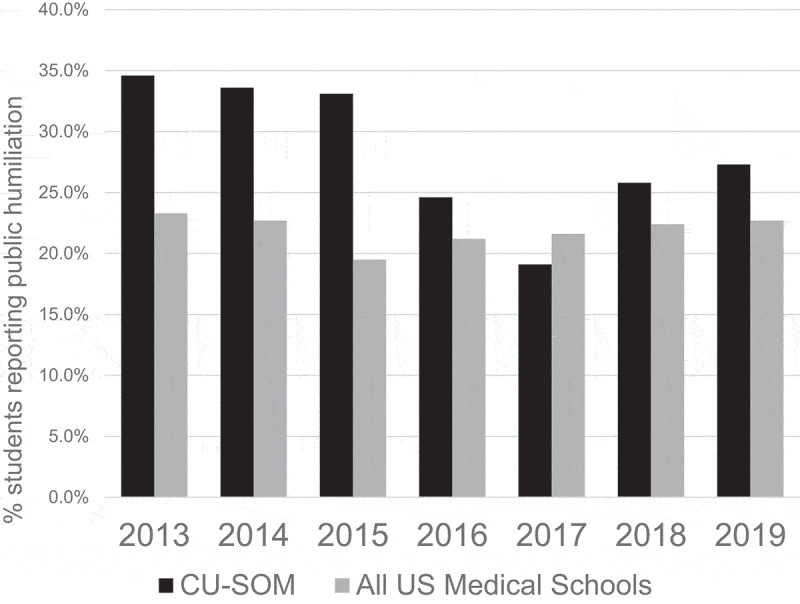
Rates of public humiliation – CU School of Medicine vs all US allopathic medical schools. Source: AAMC Graduation Questionnaire

In 2013, prior to establishing the EMTF, 62.9% of the students graduating from the CUSOM reported experiencing mistreatment, compared with 42.1% across all US medical schools. [24] In 2019, 40.3% of CUSOM students reported experiencing mistreatment, similar to the national rate (40.4%). Over the 6-year intervention period, reports of mistreatment at the CUSOM decreased by 35.9%, compared with a 4.0% decline nationally (Figure 2) [24] Student-reported public humiliation decreased from 34.6% in 2013 to 27.3% in 2019, a 21% decrease (Figure 3). ^24,242,424^ Importantly, given our focus on increasing faculty engagement and accountability, the proportion of mistreatment episodes (excluding humiliation and embarrassment) attributed to clinical clerkship faculty members decreased from 34.8% in 2013 to 18.8% in 2019, a 46% decrease. The proportion of ‘humiliation only’ mistreatment episodes attributed to clinical clerkship faculty decreased from 24.2% in 2013 to 14.4% in 2019, a 40.5% decrease.

Improvements were also noted in other areas. For example, in 2013, 88.8% of CUSOM graduates reported they ‘knew the procedures for reporting the mistreatment of medical students;’ by 2019, this percentage had increased to 94.2%. Across all US medical schools, 87.8% of the students reported knowing these procedures in 2019. [24]

**Figure 4.**
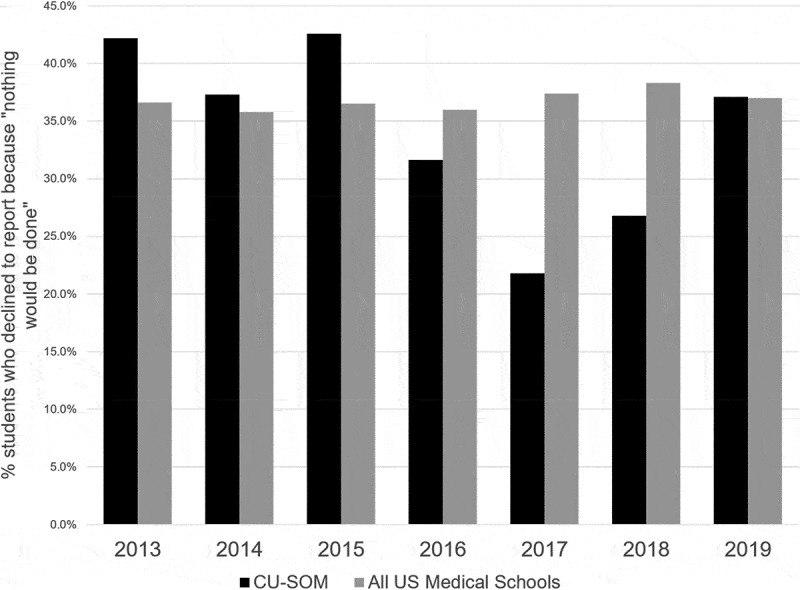
Percentage of students who, when asked ‘Why’ they did not report, did not believe that anything would be done if they were to report, CU School of Medicine vs all US allopathic medical schools. Source: AAMC Graduation Questionnaire

The EMTF and OOP also sought to increase reporting after episodes of mistreatment while also increasing students’ trust in the process and satisfaction with the outcomes. For this aim, the results were mixed. The proportion of students experiencing mistreatment who filed a report actually decreased, from 28.9% in 2013 to 24.2% in 2019; both these percentages are slightly higher than the national averages (18.7% in 2013 and 23.2% in 2019). [24] The percentage of students who declined to report mistreatment because ‘nothing would be done about it’ decreased only slightly from 42.2% in 2013 to 37.1% in 2019 (Figure 4) [14,15]. At the same, the percentage of students who declined to report mistreatment due to fear of reprisal declined from 28.9% in 2013 to 22.6% in 2019, a decrease of 22%.

## Discussion

More than twenty years ago, in a discouraging commentary, Morton *et al.* observed that ‘The dilemmas faced by medical students [are] how to survive in a threatening environment, how to please authority figures, … and how to avoid humiliation.’[16] While this is certainly not the case for most students on most days, authoritarian and hierarchal structures are still present, which can give rise to the mistreatment of students. Mistreatment may lead to burnout, stress, depressive symptoms, marginalization, cynicism and ‘moral distress’ among medical students [12–16]. In clinical settings, mistreatment also undermines teamwork, quality of care, and patient safety [1,5–12].

In order to address student mistreatment at the CUSOM, we created a unique collaboration among students, faculty members, and the medical school administration. Our task force implemented five key interventions and, over a four-year period, successfully reduced the overall rate of student mistreatment by 36% and the rate of public humiliation by 21%. The ‘Defining Mistreatment’ project helped medical students and faculty members agree on a definition of mistreatment and distinguish between mistreatment and a sub-optimal learning environment. Changes in the definition of mistreatment may have contributed to a decrease in reporting.

The faculty and administration developed a new code of conduct, an annual Faculty Promise, and an enhanced ‘teacher-learner agreement,’ and language pertaining to professional behaviors was added to faculty members’ employment contracts and annual performance reviews. The *5-Minute Clinical Orientation* addressed myths about millennial learners, reinforced students’ expectations for hard but respectful questions, and recommended ‘best practices’ to help promote a collaborative and respectful learning environment and avoid mistreatment. Four student videos were created, which helped explain mistreatment and its impact through students’ eyes. The CUSOM also established an Office of Professionalism that provided a confidential resource for reporting mistreatment and other unprofessional behaviors and a means to provide confidential and non-punitive feedback to teachers reported for mistreatment, encouraging them to reflect, empathize, apologize, and ‘get back on track.’

While these results are promising, we acknowledge that graduating medical students at our school and across the country still report mistreatment at unacceptably high rates.

### Limitations

We also acknowledge several important limitations related to this report. First, we did not implement these interventions sequentially, so it is not possible to know which interventions contributed most to the improvements we observed. The EMTF also created and distributed the *Defining Mistreatment* document, the *5-Minute Clinical Orientation*, a revised Teacher-Learner Agreement, student-created videos and other tools; however, we are unable to determine the extent to which these documents and tools were read or utilized. The CUSOM also implemented several complementary co-interventions, in addition to those specifically developed by the EMTF. For example, explicit efforts were undertaken to share mistreatment data with clerkship and residency program directors and department chairs.

Additionally, the ‘defining mistreatment’ intervention sought to clarify the definition of mistreatment and distinguish mistreatment from a sub-optimal learning environment. As noted above, changes in how mistreatment is defined may have contributed to a decrease in reporting.

We also relied on the GQ to track improvements; while the GQ response rates for CUSOM students and students nationally have been high (78–87%), not all students completed the GQ, raising the possibility of non-participation bias. We cannot determine whether students who declined to participate in the GQ are more or less likely to have experienced mistreatment. Student responses on the GQ are also subject to recall bias. In addition, at the inception of the Ending Mistreatment project, our rate of mistreatment was substantially above the national average. At least some of the improvements we observed may be the result of statistical regression to the mean.

Most importantly, these results represent a non-experimental, before–after study; caution is needed before inferring that the interventions implemented at the CUSOM were directly or solely responsible for the observed reductions in student mistreatment. Nonetheless, one strength of the current study is the inclusion of national data derived from the same measurement instrument (the GQ); this concurrent comparison group helps control for temporal trends, Hawthorne effects, instrumentation or measurement effects and other threats to the validity of the observed results.

### Ongoing challenges

As we move forward, we have identified several ongoing challenges and potential opportunities. We continue to hear from students and teachers that some degree of mistreatment is ‘part of learning’ and should be expected. We believe this perception is misguided. While hierarchal teaching and associated humiliation of students may be a traditional model for medical education, it is an ineffective and counterproductive one. Public humiliation and other forms of mistreatment have significant adverse effects on students and their future success as physicians and educators and can have a destructive impact on health-care teams and patient care. [5,7,8,31] We recognize the need to continue to educate faculty members and others about the risks inherent in a culture that tolerates mistreatment.

We must also address the need to respond to disrespectful comments about patients, family members and other health-care team members. While our original definition categorized these as indicators of a ‘suboptimal learning environment,’ they may be much closer to learner mistreatment. Dehumanizing comments against patients result in the same negative consequences as mistreatment toward medical students, including marginalization, moral distress, cynicism, erosion of professional values, and decreased quality of patient care [12,13,15,18,28]. In 2016, the GQ added a new series of questions pertaining to faculty behaviors and comments that show disrespect or lack of empathy toward patients and colleagues. In an ideal world, medical students and residents would challenge their supervisors or other team members at the moment such incidents occur; however, ‘speaking up’ in defense of humanism and professionalism presents challenges, given learners’ fears of retribution and the power differentials that persist in the clinical environment [8,24,33,40]. As a result, we believe that learning how and when to ‘speak up’ should be considered a core competency during medical training. [40] We may draw lessons from the successful implementation of incentives and protections that seek to ‘collapse medical hierarchies’ and encourage reporting of medical errors and near-misses. [10,29,37,38]

A third challenge pertains to graduate medical education (GME). While the EMTF provided a unique place at the table for medical students, until recently, we did not provide an equal voice to residents and fellows. Residents are in a unique position where they may be both victims and perpetrators of mistreatment. At the CUSOM, approximately 11% of the student mistreatment episodes are caused by an intern or resident. [24] Much like their attending supervisors, residents face an array of challenges including long work hours, lack of sleep, challenging patient encounters, inefficient workflow, and other stressors. We recognize the importance of developing GME-specific interventions and hearing the unique perspectives of these residents who are ‘caught in the middle.’[41]

There are additional challenges. We are aware that, in 2018 and 2019, the CUSOM experienced an increase in the rates of public humiliation and students not reporting mistreatment because ‘nothing will be done.’ We recognize the need for ongoing outreach to teachers and the need to track and share mistreatment data regularly. We also must provide meaningful feedback to student reporters and to the student body, to encourage reporting and reassure students that ‘something will be done’ [26,30].

There is also a need to consider faculty professionalism at the time of promotion and tenure review. While professionalism, including respectful treatment of learners, patients, and colleagues is more difficult to evaluate than research, scholarship, teaching, and other traditional measures of academic performance, a high standard of professionalism is critical to maintaining a respectful learning, clinical care and research environment. We must also develop new mechanisms to reward residents and faculty members who demonstrate exemplary professionalism, humanism, and respect for learners as a means of positive reinforcement. From a health-care system standpoint, we must strengthen collaborations with hospital medical staff, nursing and administrative leaders, in order to address workplace pressures that may result in mistreatment of learners.

Finally, students, residents and faculty members must continue to have meaningful conversations to reinforce their shared commitment to patients and to each other. We must look for new ways to celebrate respect, collaboration, the joy of clinical practice and teaching, and the ‘collegiality of kindred spirits.’ [14]

## Conclusion

We have outlined a series of interventions implemented at one medical school by a collaborative task force that included students, faculty members, and administrative leaders. These interventions were associated with a sharp reduction in the rates of mistreatment reported by graduating medical students. The collaborations and open dialogues between student and faculty leaders helped each party learn from the other, as we identified unexpected misunderstandings and challenges. The collaboration reinforced our shared commitment to upholding a respectful learning environment and ending mistreatment.

We have made important progress. However, this work is not complete. Continued collaboration among students, residents, faculty, and the administration will be required if we are to reach the goal of ending mistreatment.
